# Hypoxic exposure can improve blood glycemic control in high-fat diet-induced obese mice

**DOI:** 10.20463/pan.2020.0004

**Published:** 2020-03-31

**Authors:** Yeram Park, Inkwon Jang, Hun-Young Park, Jisu Kim, Kiwon Lim

**Affiliations:** 1 Department of Physical Education in Graduated school, Konkuk University, Seoul Republic of Korea; 2 Department of Sports Medicine and Science in Graduated School, Konkuk University, Seoul Republic of Korea; 3 Physical Activity and Performance Institute (PAPI), Konkuk University, Seoul Republic of Korea

**Keywords:** Hypoxia, OGTT, insulin, diabetes, HOMA-IR, blood glucose, obesity

## Abstract

**[Purpose]:**

Blood glucose and insulin resistance were lower following hypoxic exposure in previous studies. However, the effect of hypoxia as therapy in obese model has not been unknown.

**[Methods]:**

Six-week-old mice were randomly divided into chow diet (n=10) and high-fat diet (HFD) groups (n=20). The chow diet group received a non-purified commercial diet (65 % carbohydrate, 21 % protein, and 14 % fat) and water ad libitum. The HFD group was fed an HFD (Research Diet, #D12492; 60% kcal from fat, 5.24 kcal/g). Both groups consumed their respective diet for 7 weeks. Subsequently, HFD-induced mice (12-weeks-old) were randomly divided into two treatment groups : HFD-Normoxia (HFD; n=10) and HFD-Hypoxia (HYP; n=10, fraction of inspired=14.6%). After treatment for 4 weeks, serum glucose, insulin and oral glucose tolerance tests (OGTT) were performed.

**[Results]:**

Homeostatic model assessment values for insulin resistance (HOMA-IR) of the HYP group tended to be lower than the HFD group. Regarding the OGTT, the area under the curve was 13% lower for the HYP group than the HFD group.

**[Conclusion]:**

Insulin resistance tended to be lower and glucose uptake capacity was significantly augmented under hypoxia. From a clinical perspective, exposure to hypoxia may be a practical method of treating obesity.

## INTRODUCTION

Worldwide more than 1.4 billion adults are overweight, of which more than 400 million are obese^1^. Obesity represents a major health burden because it is accompanied by an increased risk of insulin resistance (IR) and diabetes.

Recent data points to an important role of hypoxia in glucose metabolism disorders^2-3^. Hypoxia is a state of low oxygen consumption and can be classified into four stages based on altitude. Low hypoxia is defined as an altitude of 500-2,000 m (inspired oxygen fraction (F_I_O_2_) = 16.7-19.8%), moderate hypoxia is 2,000-3,000 m (F_I_O_2_ = 14.8-16.7%), high hypoxia is 3,000-5,500 m (F_I_O_2_ = 10.9-14.8%) and extreme hypoxia as >5,500 m (F_I_O_2_ < 10.9%)^4^.

High altitude populations have lower blood glucose concentrations and a lower incidence of type 2 diabetes. Hill et al.^5^ demonstrated that blood glucose concentration and insulin resistance were lower following gradual ascent in altitude (3,600-5,120 m). Woolcott et al.^6^ reported that prolonged exposure to high hypoxia (altitude 3,500 m) might decrease concentration. Wang et al.^2^ and Mackenzie et al.^7^ reported that exposure to high-intermittent hypoxia (inspired oxygen fraction=14.6-14.7%) improved glycemic control. Along with glycemic control, the capacity for glucose uptake is important. Hypoxia itself stimulates glucose uptake mediated by AMP-activated protein kinase (AMPK) and glucose transporter 4 (GLUT4) translocation^8-9^.

High hypoxia, which is effective in regulating blood glucose concentration, has the potential to be a therapeutic method for obesity-related glucose metabolic disorders. However, in the case of obese model studies, most studies have used hypoxia with extreme intensity as a method to induce obstructive sleep apnea (OSA)^10-11^.

Taken together, it is necessary to confirm the effect of high hypoxia on an obese model, which has a higher risk of glucose metabolic disorders. Therefore, the purpose of this study is to investigate the effects of high hypoxia on high-fat diet (HFD)-induced obese mice.

## METHODS

### Animal Subjects

Five-week-old male ICR mice were purchased from Orient Bio Inc. (Seongnam, Korea). All mice were housed in standard plastic cages under controlled humidity (50%) and temperature (23 ± 1℃) conditions in a 12 h/12 h dark/light cycle. Mice were acclimatized to the laboratory housing condition for 1 week. The animal experiments were conducted according to protocols reviewed and approved by the Konkuk University Institutional Animal Care and Use Committee (permit number: KU19187). The chow diet mice received a non-purified commercial diet (65% carbohydrate, 21% 86 protein, and 14% fat) and water ad libitum. The HFD mice were fed with an HFD (Research Diet, #D12492; 60% kcal from fat, 5.24 kcal/g) from 5 weeks old.

### Animal Model Preparation

**Figure 1.**
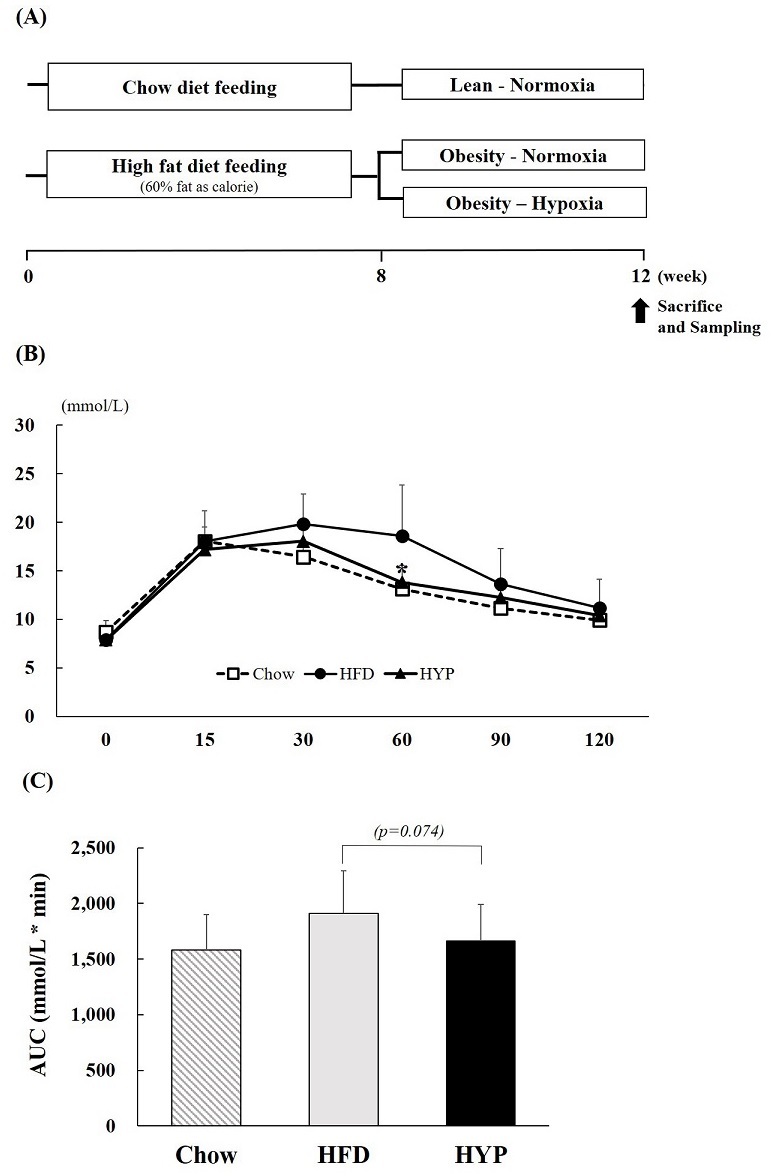
(A) Experiment design (B) OGTT and (C) the area under the curve responses to an oral glucose challenge (2 g/kg) after 12 h of food deprivation at week 12. Values represent the mean ± S.D. (n = 10). Chow (□), mice consuming regular diet; HFD (●), mice consuming high-fat diet; HYP (▲), mice fed with high-fat diet in exposure of hypoxia. *p < 0.05 vs. HFD.

Six-week-old mice were randomly divided into two groups: chow diet (n=10) and HFD groups (n=20). Both groups consumed each diet for 7 weeks. After the 7 weeks, the HFD-induced mice (12-weeks-old) were randomly divided into two treatment groups: HFD-Normoxia (HFD; n=10), HFD-Hypoxia (HYP; n=10). In addition, the chow diet group consumed a chow diet for the experiment period (Chow; n=10) (Fig. 1A).

### Hypoxic exposure

The HYP group was placed in identical commercially designed chambers (model HCC-550, SFET, Korea) and exposed to hypoxic conditions during only the light phase (from 19:00 hours to 07:00 hours) to coincide with the mouse sleep cycle. Oxygen concentration was set at 14.6% (although the actual oxygen concentration raged from 14.4-14.7% during the experiment). The use of the high-altitude intervention in our study was with reference to previous reports in the literature^2,7^. After the 4-week exposure, all tissue harvesting was performed. Food was withdrawn for 4 h during the day of tissue harvesting. Mice were anesthetized with avertin.

### Oral glucose tolerance test (OGTT)

An oral glucose tolerance test (OGTT) was performed at the end of the treatment. On the test day, animals were fasted for 12 h, after which glucose (2 g/kg) was orally administered. Blood samples were taken from the tail at 0, 15, 30, 60, 90, and 120 min after glucose administration. The level of glucose was measured using a glucose meter (ACCU Check, France). The areas under the curve (AUCs) were calculated using trapezoidal integration.

### Measurement of blood glucose and insulin

Serum was harvested from ICR mice at the end of the experiment and stored at −80°C. Insulin levels were measured with a mouse enzyme-linked immunosorbent assay (ELISA) kit according to the manufacturer’s instructions (Alpco, Salem, NH). Glucose levels were determined using a glucose meter (ACCU Check, Penzberg, Germany).

### Statistical analysis

Significant differences between groups were determined using independent t-tests (SPSS for Windows, version 24.0, Chicago, IL, USA). All values are reported as a mean ± S.D. A p-value of < 0.05 was considered statistically significant.

## RESULTS

### Body weights and food consumption

The body weights and food consumption are shown in Table 1. There was a significant difference between the initial and final body weight in the HYP group. The food consumption was not different between the HYP and HFD groups.

**Table 1.** The change of body weight, food intake and blood variables

	Chow	HFD	HYP
Initial body weight(g)	39.7 ± 2.9	50.1 ± 5.2	51.2 ± 3.8
Final body weight(g)	38.1 ±1.6	48.2 ± 3.6	48.3 ± 2.3^†^
Body weight gain(g)	-1.6 ± 1.6	-1.9 ± 3.6	-3.1 ± 2.3
Food intake(g/day)	4.4 ± 0.4	3.2 ± 0.4	3.3 ± 0.6
Serum glucose(mmol/L)	10.7 ± 2.1	12.7 ± 1.4	13.0 ± 1.3
Serum Insulin(μU/mL)	1.9 ± 0.0	2.0 ± 0.1	1.9 ± 0.0
HOMA-IR	0.9 ± 0.2	1.1 ± 0.1	1.1 ± 0.1

Values represent the mean ± S.D. (*n* = 10). Homeostasis Model Assessment was used to calculate an index of insulin resistance as insulin (μU/mL) × glucose (mmol/L)/22.5. † p < 0.05 vs. initial.

### Serum glucose, insulin, and insulin resistance index

Fasting serum glucose and insulin levels were measured at the end of the treatment. Hyperglycemia did not develop in any of the groups over the 4-week trial (Table 1). Serum insulin levels tended to be lower in the HYP group compared to the HFD group (p=0.057). The homeostatic model assessment values for insulin resistance (HOMA-IR), calculated by insulin (μU/ml) × glucose (mM)/22.5^12^, of the HYP group tended to be lower than the HFD group.

### Oral glucose tolerance test (OGTT)

After 4-weeks of hypoxic exposure, the OGTT was performed. Glucose challenge dramatically increased the blood glucose levels in HFD fed mice compared to those in the HYP group. HYP treated groups were seen to have significantly reduced rising blood glucose levels, especially at 60 min time points, which were at similar levels to the chow diet-fed group (Fig. 1B). When the area under the curve (AUC) was compared between groups, HYP treated groups showed a 13% lower AUC compared to the HFD control group (Fig. 1C).

## DISCUSSION

We investigated the effects of hypoxic exposure on glycemic control in HFD-induced obese mice. We found that fasting blood insulin tended to be lower in the hypoxic exposed group (HYP) than the normoxia group (HFD). Insulin sensitivity, determined according to the HOMA-IR, tended to be higher in the HYP group than in the HFD group.

In addition, as shown in the results related to the OGTT, blood glucose concentration was lower in the HYP group and was a similar level to the chow diet-fed group. Therefore, these results implied that hypoxic exposure could improve glycemic control.

As control factors of glycemic control, changes in glucose transporters and its translocation impact glucose homeostasis. Hypoxia causes an increase in glucose transporters into the skeletal muscle. Primarily, insulin-dependent glucose uptake in skeletal muscle is mediated by the translocation of GLUT-4 transporter because of increased activation of the PI3K/Akt pathway^13^. Wang et al.^14^ reported that GLUT-4 protein expression was 33% higher in the skeletal muscle following hypoxic exposure (fractional inspired O2 of 15%) for 4 weeks in mice. Besides, a previous study reported that blood glucose concentration and insulin resistance were lower following gradual ascent in altitude (3,600-5,120 m)^5^. In the present study, blood insulin tended to be lower, and glucose concentration was lower in the hypoxic exposure group. These results might be due to mediation by a glucose transporter. Further studies on protein expression of glucose transporters and glucose uptake are required.

In addition to hypoxic therapy, previous studies suggest that regular exercise reduces the risk of developing type 2 diabetes and significantly improves glycemic control^15-20^. This might be due to the translocation of glucose trans¬porters (e.g., GLUT-4)^21-22^. In¬deed, studies have demonstrated increased GLUT-4 concentrations with aerobic training, which is accompanied by an increase in insulin-mediated glucose uptake^23-25^.

Moreover, insulin-mediated glucose uptake is inversely correlated with body fat mass^26-27^. In a previous study, hypoxic exposure (altitude 2,200-4,000 m) combined with aerobic exercise decreased body weight and fat mass^28-30^. Therefore, it could be used as a therapy for obesity with glucose control disorders. In the present study, we confirmed that hypoxic exposure decreased body weight and blood glucose levels. We implied that this was induced by reduced body fat mass. Therefore, exercise, in combination with hypoxic exposure, could have a synergic effect on glucose control within a shorter period than exercise alone.

In conclusion, insulin resistance tended to be lower, and glucose uptake capacity was significantly augmented under high hypoxia. From a clinical perspective, intermittent exposure to high hypoxia (14.6 F_I_O_2_) may be a practical method for treating obesity. Further, a combination of exercise could have a synergic effect. However, the current study did not confirm the protein expression of glucose metabolism. Therefore, future research should be conducted to elucidate the molecular mechanism of glucose metabolism.

